# Dentists' Convention

**Published:** 1898-06

**Authors:** 


ARTICLE VII.

### DENTISTS' CONVENTION.

SECOND UNION MEETING OF MARYLAND, WASHINGTON AND VIRGINIA ASSOCIATIONS.

AN UP TO DATE EXHIBIT.

**Interesting Operations Practically Demonstrating New Methods Methods of Treatment, Together with the Unique Devices for Carrying Them Out.**

The second tri-union meeting of the Maryland State, Washington City and Virginia State Dental Associations, held for the first time in Baltimore, began yesterday morning in the dental department of the Baltimore Medical College, Linden avenue and Howard street.

Over four hundred dentists from all sections of the Union were present. They thronged the big building and wandered about among the apparatus and appliances that were on all three floors. The outside of the building is draped with red, white and blue colors, and over the door is an immense sign of "Welcome." A general air of good fellowship prevails among the dentists.

The arrangements for the display were made by Drs. George R. Carter and Wallace W. Bruce and are excellently planned. The large room on the first floor is given over to the exhibit of the S. S. White Company, who have a fine display of electrical dental engines and pluggers. The famous Wilkerson chair, invented by Dr. B. M. Wilkerson, of Baltimore, is also on exhibition for the first time and is attracting much comment.

### INSTRUMENTS OF ALL KINDS.

Upstairs the two front rooms fairly swarm with deadly looking instruments of all sizes, shapes and kinds, and all equally unprepossessing in appearance to an outside spectator. There is a great display of artificial teeth, covering two large tables, and here the dentists rise to the highest pitch of enthusiasm, as remarkable progress is said to have been made along this line. Every conceivable kind of an instrument that could be used by a dentist is on exhibition. Twenty-five of the big Eastern firms are represented.

Dr. William A. Mills, chairman of the joint executive committee, called the meeting to order in the big assembly hall at half past 9 o'clock in the morning. Presidents F. F. Drew, W. N. Cogan and J. V. Haller, of the Maryland, Washington and Virginia associations, respectively, were seated on the platform with Dr. Mills. Rev. Peregrine Wroth, rector of the Protestant Episcopal Church of the Messiah, opened the meeting with prayer. Mr. Wroth prayed that the present war with Spain would speedily be settled in the right way and that peace would be restored.

### WELCOMED THE DELEGATES.

President Drew made an address welcoming the visiting delegates to Baltimore, and was thanked by Drs. Cogan and Haller. Dr. Mills then presented President Drew with a gavel, remarking, as he did so, that the gavel was made out of the wood taken from the mulberry tree at St. Mary's parish, St. Mary's county, under which Lord Baltimore signed his treaty of peace with the Indians. Dr. Mills said that he hoped President Drew would not be called to use the gavel in any but peace proceedings during the convention.

Dr. Drew, after disposing of some routine business, called upon Dr. A. W. Sweeney, of Washington, to read his paper on "Dental Laws and What They Have Accomplished." Dr. J. N. Crouse then read a paper on "Patents and Patent Abuses." Dr. W. G. Chase, of Philadelphia, who was to have read the first paper, "The Status of the Degree D. D. S. Under the Law," read it later.

There were short debates over each paper, the speakers being limited to five minutes each. The morning session adjourned at 12 o'clock, and after luncheon the afternoon session opened.

### PERFORMED OPERATIONS.

The entire afternoon was devoted to clinics, and a number of interesting operations were performed by some of the best known dentists. Dr. George E. Milholland, of Baltimore, was the first demonstrator, and spoke on electroplating without battery, using the Pohlman method and fluid.

Others were Dr. David Genese, exhibiting an office lamp using acetyline gas; Drs. H. M. Schooley and W. E. Diffenderfer, of Washington; Dr. F. S. Condit, of Chicago, combination plate and bridge; Dr. T. D. Shamway, Plymouth, Mass., filling of gold and tin by affinity; Dr. S. Lua Syckes, of Cumberland, Md., anchoring cohesive gold in cement and tin foil, Dr. William L. Fish, of Newark, N. J., device for holding the teeth in position when loosened or when implanted.

Dr. William B. Finney, of Baltimore, the Finney method of placing gold tips on the cutting edges of the anterior teeth to prevent cracking of the same. Drs. James

G. Palmer, of New York, William Donnelly, of Washington, and T. S. Waters, of Baltimare, also demonstrated new theories.

PAPERS ON TECHNICAL SUBJECTS.

The evening session was held in the lecture hall of the Baltimore Medical College. President Drew was in the chair, and the following papers were read and discussed; Dr. C. W. Mitchell, of Baltimore, "Injurious Effects of Artificial Food Upon Deciduous Teeth;" Dr. H. O. Reik, of Baltimore, "Sterilization of Instruments;" Dr. H. Jerome Allen, of Wasnington, "The Use of Formalin;" Dr. H. S. Harvey, of Battle Creek; Mich., "The Chemico-Micro-Organisms of the Stomach in Relation to Diseases of the Oral Cavity;" Dr. W. Xavier Sudduth, of Chicago, "Drug Habits Among Professional People."

In the evening the dentists banqueted at the Eutaw House. The banquet committee were Drs. B. Holly Smith, William A. Montell and W. W. Dunbracco.

The officers of the Maryland State Dental Association are: President, Dr. F. F. Drew; vice-presidents, Drs. M. G. Sykes and E. E. Cruzon; recording secretary, Dr. George E. Hardy; corresponding secretary, Dr. W. W. Dunbracco; treasurer, Dr. S. C. Pennington.

